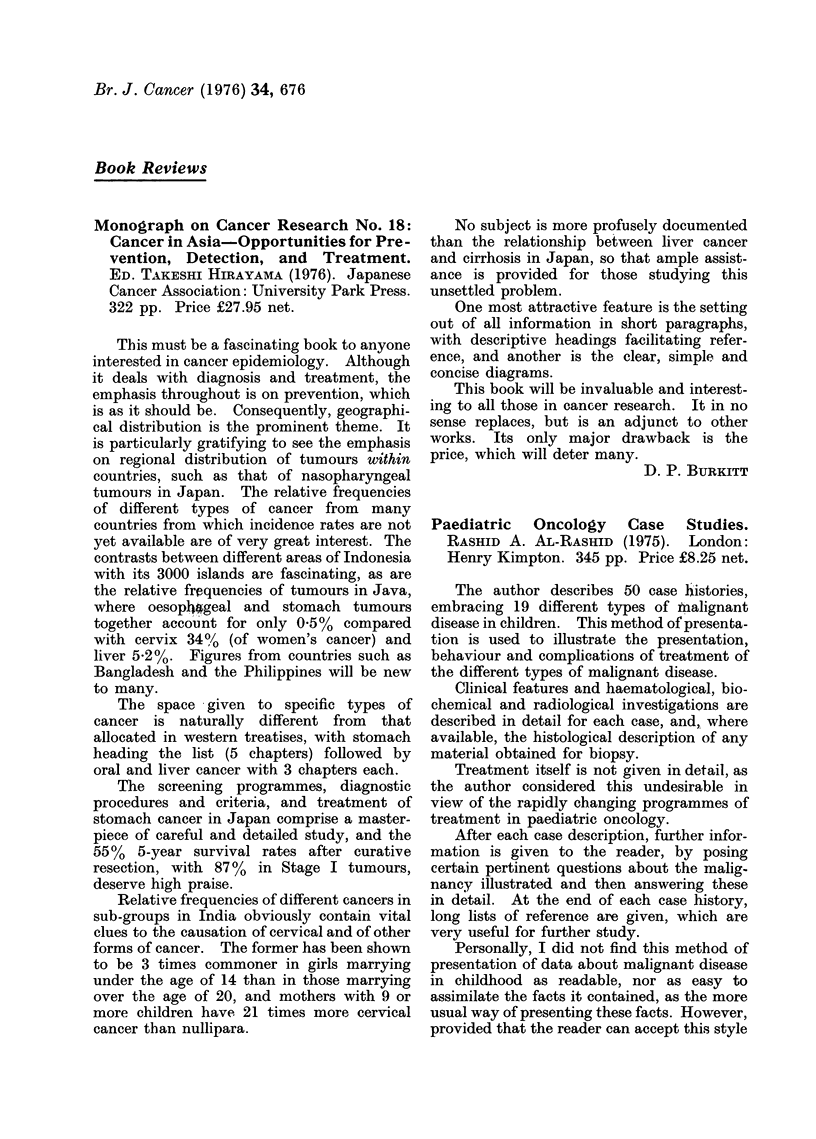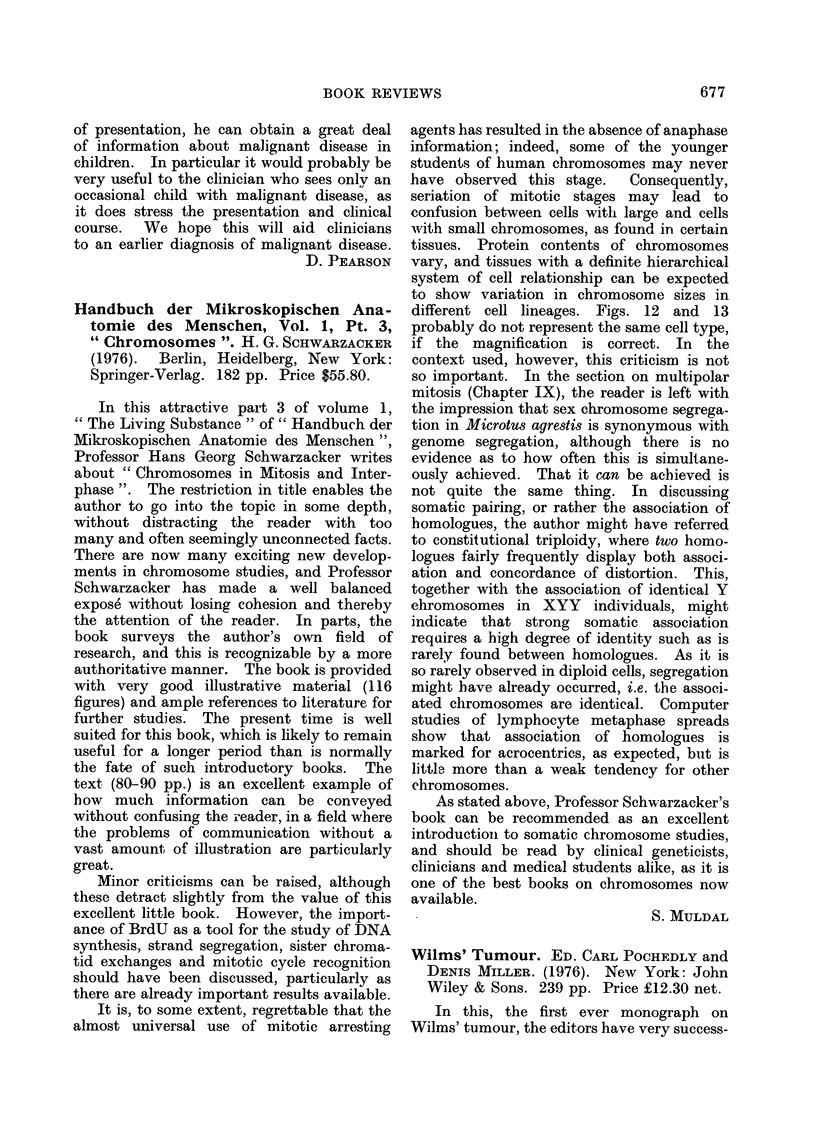# Paediatric Oncology Case Studies

**Published:** 1976-12

**Authors:** D. Pearson


**Paediatric Oncology Case Studies.** RASHID A. AL-RASHID (1975). London: Henry Kimpton. 345 pp. Price £8.25 net.

The author describes 50 case histories, embracing 19 different types of malignant disease in children. This method of presentation is used to illustrate the presentation, behaviour and complications of treatment of the different types of malignant disease.

Clinical features and haematological, biochemical and radiological investigations are described in detail for each case, and, where available, the histological description of any material obtained for biopsy.

Treatment itself is not given in detail, as the author considered this undesirable in view of the rapidly changing programmes of treatment in paediatric oncology.

After each case description, further information is given to the reader, by posing certain pertinent questions about the malignancy illustrated and then answering these in detail. At the end of each case history, long lists of reference are given, which are very useful for further study.

Personally, I did not find this method of presentation of data about malignant disease in childhood as readable, nor as easy to assimilate the facts it contained, as the more usual way of presenting these facts. However, provided that the reader can accept this style of presentation, he can obtain a great deal of information about malignant disease in children. In particular it would probably be very useful to the clinician who sees only an occasional child with malignant disease, as it does stress the presentation and clinical course. We hope this will aid clinicians to an earlier diagnosis of malignant disease.

D. PEARSON